# Inactivation of the Basolateral Amygdala to Insular Cortex Pathway Makes Sign-Tracking Sensitive to Outcome Devaluation

**DOI:** 10.1523/ENEURO.0156-22.2022

**Published:** 2022-09-28

**Authors:** Sara E. Keefer, Daniel E. Kochli, Donna J. Calu

**Affiliations:** Department of Anatomy and Neurobiology, University of Maryland School of Medicine, Baltimore, MD 21201

**Keywords:** basolateral amygdala, behavioral flexibility, individual differences, insular cortex, outcome devaluation

## Abstract

Goal-tracking (GT) rats are sensitive to Pavlovian outcome devaluation while sign-tracking (ST) rats are devaluation insensitive. During outcome devaluation, GT rats flexibly modify responding to cues based on the current value of the associated outcome. However, ST rats rigidly respond to cues regardless of the current outcome value. Prior work demonstrated disconnection of the basolateral amygdala (BLA) and anterior insular cortex (aIC) decreased both GT and ST behaviors. Given the role of these regions in appetitive motivation and behavioral flexibility, we predicted that disrupting BLA to aIC pathway during outcome devaluation would reduce flexibility in GT rats and reduce rigid appetitive motivation in ST rats. We inhibited the BLA to aIC pathway by infusing inhibitory DREADDs (hM4Di-mcherry) or control (mCherry) virus into the BLA and implanted cannulae into the aIC to inhibit BLA terminals using intracranial injections of clozapine N-oxide (CNO). After training, we used a within**-**subject satiety-induced outcome devaluation procedure in which we sated rats on training pellets (devalued condition) or homecage chow (valued condition). All rats received bilateral CNO infusions into the aIC before brief nonreinforced test sessions. Contrary to our hypothesis, BLA-IC inhibition did not interfere with devaluation sensitivity in GT rats but did make ST behaviors sensitive to devaluation. Intermediate rats showed the opposite effect, showing rigid responding to cues with BLA-aIC pathway inactivation. Together, these results demonstrate BLA-IC projections mediate tracking-specific Pavlovian devaluation sensitivity and highlights the importance of considering individual differences in Pavlovian approach when evaluating circuitry contributions to behavioral flexibility.

## Significance Statement

Individual differences in sign-tracking (ST) and goal-tracking (GT) behavior are characterized by differences in motivational properties toward reward predictive cues, which can predict differences in behavioral flexibility and addiction-related behaviors. Goal-trackers flexibly adjust their behavior when the value of outcomes change, while sign-trackers rigidly respond to cues after devaluation of the outcome. Preclinical research indicates neurobiological differences between ST and GT individuals, resulting in behavioral differences before drug use and addiction. The current study tested the hypothesis that tracking-specific differences in utilization of an amygdala-cortical circuitry contributes to behavioral flexibility differences. This work ultimately furthers our understanding of the behavioral and neurobiological underpinnings of individual differences in adaptive behaviors and addiction vulnerability.

## Introduction

Substance use disorder (SUD) only affects a small portion of individuals who engage in drug use. Individuals with SUD chronically relapse into compulsive drug seeking and drug taking despite negative consequences and are less likely to change their behavior despite environmental pressures. In preclinical models, addiction vulnerability is examined with sign-tracking (ST) and goal-tracking (GT) phenotypes defined in a Pavlovian lever autoshaping (PLA) procedure (Hearst and Jenkins, 1974; Flagel et al., 2009). ST rats approach and vigorously engage with an insertable lever cue, a behavior that remains rigid when the associated reward is devalued. GT rats approach and engage with the food cup during the lever cue, a behavior that flexibly decreases when the associated reward is devalued (Morrison et al., 2015; Nasser et al., 2015; Patitucci et al., 2016; Smedley and Smith, 2018; Amaya et al., 2020; Keefer et al., 2020). Here, we examine a brain pathway that is implicated in both appetitive motivation and behavioral flexibility to determine its contribution to flexibility differences in GT and ST rats.

The basolateral amygdala (BLA) is involved in both incentive learning and motivation (Hatfield et al., 1996; Johnson et al., 2009; Chang et al., 2012; Wassum and Izquierdo, 2015), and its projections to the anterior insular cortex (aIC) are necessary for both GT and ST behaviors (Nasser et al., 2018). Contralateral disconnection of the BLA and aIC decreases GT approach and increases the latency to both goal-track and sign-track. Another study showed temporally specific involvement for the BLA and aIC during instrumental outcome devaluation, with the BLA necessary for encoding the degraded outcome value and the aIC necessary for the retrieval of that outcome value at test (Parkes and Balleine, 2013). These results indicate information flow from the BLA to aIC is necessary in behavioral flexibility. Similarly, communication between the BLA and the orbitofrontal cortex (OFC), which borders the aIC, is critical for behavioral flexibility across species (Baxter et al., 2000; Fiuzat et al., 2017) and direct BLA to OFC projections are necessary for Pavlovian, but not instrumental, outcome devaluation (Lichtenberg et al., 2017). These findings indicate communication from the BLA to aIC is necessary for GT behaviors and for behavioral flexibility in instrumental outcome devaluation (for review, see Keefer et al., 2021).

The current study first examines whether communication from the BLA to aIC is necessary for Pavlovian outcome-specific satiety devaluation. Then, we wanted to determine the extent to which tracking-specific pathway utilization mediates GT and ST differences in devaluation sensitivity. We hypothesized that intact GT rats would be devaluation sensitive and that chemogenetic inhibition of the BLA to aIC pathway would make GT rats devaluation insensitive (Morrison et al., 2015; Nasser et al., 2015; Amaya et al., 2020; Keefer et al., 2020; Kochli et al., 2020). Furthermore, we hypothesized that intact ST rats would be devaluation insensitive and that chemogenetic inhibition of the BLA to aIC pathway would generally reduce ST (Nasser et al., 2018) or potentially make them devaluation sensitive (Nasser et al., 2015; Amaya et al., 2020; Keefer et al., 2020; Kochli et al., 2020). To inactivate the direct pathway from BLA to aIC, we expressed inhibitory chemogenetic constructs into bilateral BLA and implanted bilateral guide cannulae into the aIC to directly inhibit BLA terminals in aIC during outcome-specific satiety devaluation.

## Materials and Methods

### Subjects

Male and female Long–Evans rats (Charles River Laboratories; approximately eight weeks of age on arrival; *N* = 160 run as 5 cohorts) were maintained on a 12/12 h light/dark cycle with lights off at 9 A.M. Rats were doubled-housed on arrival with *ad libitum* access to standard laboratory chow and water, and single-housed housed after acclimation and before surgery or behavioral procedures. We surgerized two cohorts of rats before all behavioral training and testing and surgerized three cohorts after determining tracking phenotype but before devaluation testing. (Results did not differ regardless of surgery and behavioral timeline.) We performed all behavioral procedures during the dark phase on the cycle. During all behavioral training and testing, we food-restricted rats to ∼90% of their maximum achieved body weight. We conducted all experiments in accordance to the *Guide for the Care and Use of Laboratory Animals* (8th edition, 2011, National Research Council) and were approved by University of Maryland, School of Medicine Institutional Animal Care and Use Committee (IACUC).

### Surgical procedures

We anesthetized rats with isoflurane (Vetone; 5% induction, 1–3% maintenance throughout surgery). We placed rats in a stereotaxic apparatus (model 900, David Kopf Instruments) and maintained rats body temperature with a heating pad throughout surgery. We administered subcutaneous injection of carprofen analgesic (5 mg/kg) and a subdermal injection of the local anesthetic lidocaine (10 mg/ml) at the incision site before first incision. We leveled the skull by leveling bregma and lambda on the dorsal-ventral plane and performed craniotomies above each injection site with a drill. We used a 10 μl Hamilton syringe (Hamilton) to deliver the virus into bilateral BLA using the following coordinates: AP −2.8 mm, ML ± 5.0 mm, DV −8.5 mm 0° from midline relative to bregma surface. We infused 600 nl of AAV8-hSyn-hM4Di-mCherry (hM4Di) or AAV8-hSyn-mCherry (mCherry; Addgene) into each BLA via a micropump (UltraMicroPump III, World Precision Instruments) at a rate of 250 ml/min and the syringe was left in place for 10 min after infusion to allow for viral diffusion. After closing BLA craniotomies with bone wax, we implanted 23-gauge guide cannulae bilaterally 1 mm above our target region in the aIC using the following coordinates: AP +2.8 mm, ML ± 4.0 mm, DV −4.8 mm 0° from midline relative to bregma surface and anchored them with jeweler’s screws and dental cement. We inserted obturators into the guide cannulae, which were removed periodically throughout recovery and training to ensure patency. We moved rats to a recovery cage on a heating pad, administered carprofen analgesic (5 mg/kg, s.c.) at 24 and 48 h postsurgery, and monitored their health until behavioral procedures.

### Apparatus

We conducted behavioral experiments in identical behavioral chambers (25 × 27 × 30 cm; Med Associates) located in a different room than the colony room. Each chamber was contained in individual sound-attenuating cubicles with a ventilation fan. On one wall, a red house light (6 W) was illuminated during PLA sessions and devaluation tests. The opposite wall had a receded food cup with photobeam detectors, and the cup was 2 cm above the grid flood. A programmed pellet dispenser was attached to the food cup and delivered 45-mg food pellets [catalog #1811155; Test Diet Purified Rodent Tablet (5TUL); protein 20.6%, fat 12.7%, carbohydrate 66.7%]. One retractable lever was located 6 cm above the grid floor on either side of the food cup, and lever side was counterbalanced between subjects.

### Pavlovian Lever Autoshaping

Before training, we habituated rats to the food pellets in their home cage to reduce novelty to the food. Next, we trained rats on daily PLA sessions that lasted ∼26 min and included 25 lever presentations (conditioned stimulus; CS) and occurred on a VI 60-s schedule (50–70 s). After the 10-s lever presentation, the lever retracted and two 45 mg food pellets were delivered via food cup, noncontingent on the rat’s behavior. After 25 trials, the red house light turned off, and we returned rats to their cage and colony room.

### Satiety-induced outcome devaluation testing

After the 5th PLA session, we gave rats two sessions of satiety-induced outcome devaluation testing. Rats had 1 h of access to 30 g of either their homecage chow (valued condition) or food pellets used during PLA training (devalued condition) in a prehabituated ceramic ramekin. Within 15 min after the end of the satiation hour, we infused 0.25 μl of 1 mm clozapine N-oxide (CNO; Tocris or Abcam) dissolved in aCSF into bilateral aIC over 1 min and left injectors in place for an additional minute to allow to diffusion of solution. We waited 10–15 min after infusion to allow binding of the CNO to the DREADD receptors, and then placed rats into the behavioral chambers for devaluation probe test. Tests consisted of 10 nonreinforced lever presentations on VI 60-s schedule (50–70 s). After test, we gave rats a 30-min food choice test in their homecage, which included 10 g of chow and 10 g of food pellets in separate ramekins to confirm satiety was specific to the outcome they were prefed.

### Behavioral measurements

For PLA training sessions and devaluation probe tests, we recorded number and duration of contacts, latency to contact, and probability of contact for each behavior to the food cup and the lever during the 10-s CS (lever) period. On trials with no contact, a latency of 10 s was recorded. Probability of contact was calculated by determining the number of trials that the lever or food cup contact was made, divided by total number of trials in that session.

To determine tracking phenotype, we used a Pavlovian conditioned approach (PCA) analysis (Meyer et al., 2012) which quantifies the continuum of lever-directed (ST) and food cup-directed (GT) behaviors. PCA scores are the average of three separate score measures: (1) preference score, (2) latency score, and (3) probability score. Preference score is number of lever contacts minus number of food cup contacts during the CS divided by the sum of these two measures. Latency score is the average time to make a food cup contact minus the time to make a lever contact during the CS divided by 10 s (the duration of the CS). Probability score is the probability to make a lever contact minus probability to make a food cup contact across trials in a session. PCA score for each rat was determined by averaging the PCA scores for PLA sessions 4 and 5. ST PCA range from +0.25 to +1.0, GT PCA range from −0.25 to −1.0, and intermediate PCA range from −0.24 to +0.24.

For devaluation probe tests, we examined total behavior (sum of food cup and lever contacts during the 10-s CS period) and responding of each behavior separately. We also examined latency to respond. For consumption data on test days, we recorded the amount of pellets and chow in grams during satiety hour and during the 30 min choice test.

### Histology

After all behavioral training and testing finished, we anesthetized rats with isoflurane and transcardially perfused with 100 ml of 0.1 m PBS then 400 ml of 4% paraformaldehyde in 0.1 m sodium phosphate, pH 7.4. We extracted brains and postfixed them in the 4% paraformaldehyde solution for at least 2 h before incubation in a 30% sucrose in 0.1 m sodium phosphate for at least 24 h at 4°C. We rapidly froze brains in dry ice and stored them in −20°C until slicing. Using a cryostat (Leica Microsystems), we collected 30-μm sections into four series through the cannulae placement in aIC and through the virus infusion sections in the BLA. Sliced tissue was stored in cryopreservant in −20°C until mounting or immunohistochemistry. We mounted cannulated aIC sections onto gelatin-coated slides, and after drying, we stained with cresyl violet, coverslipped with Permount, and examined under a light microscope for confirmation of cannulae placement into the aIC. We mounted BLA sections onto SuperFrost slides, and after drying, we coverslipped with Vectashield mounting medium with DAPI. We used immunohistochemistry to amplify hM4Di-mCherry expression on the terminals in the aIC for confirmation of terminal expression. Floating aIC sections were rinsed in 0.1 m PBS two times for 10 min and blocked for 1 h with 2% normal goat serum and 0.3% Triton X-100 in PBS. Sections were rinsed in PBS twice for 10 min and incubated in blocking solution with anti-dsRed primary antibody raised in rabbit (1:500; Takara Bio catalog #632496, RRID:AB_10013483) overnight in 4°C with gentle agitation. Sections were rinsed two times for 10 min each in the blocking solution, then incubated in blocking solution containing AlexaFluor-594 goat anti-rabbit (1:500; Invitrogen). After three 10-min rinses in PBS, we mounted sections onto SuperFrost slides and coverslipped with Vectashield mounting medium with DAPI. We confirmed viral expression in the BLA and in terminals within the aIC under 5× or 10× using a Confocal SP8 (Leica Microsystems) and used anatomic boundaries defined by previously published work (Paxinos and Watson, 2007; Swanson, 2004). We excluded rats if cannulae or viral placements were outside the region of interest (Fig. 4), or if terminal expression could not be confirmed, which resulted in *N* = 75 (37 females, 38 males): GT: 13 mCherry, 12 hM4Di; ST: 13 mCherry, 13 hM4Di; and INT: 13 mCherry, 11 hM4Di.

### Statistical analysis

We analyzed data using SPSS statistical software (IBM v.25). We used mixed-design repeated measures ANOVAs. When applicable, the within-subject factors were response (food cup, lever) and outcome value (valued, devalued), and the between-subject factors were virus (hM4Di, mCherry), tracking group (ST, INT, GT), and sex (female, male). Significant main effects and interactions were followed by *post hoc* paired samples or independent *t* tests.

## Results

### Limited Pavlovian Lever Autoshaping

**Figure 1.**
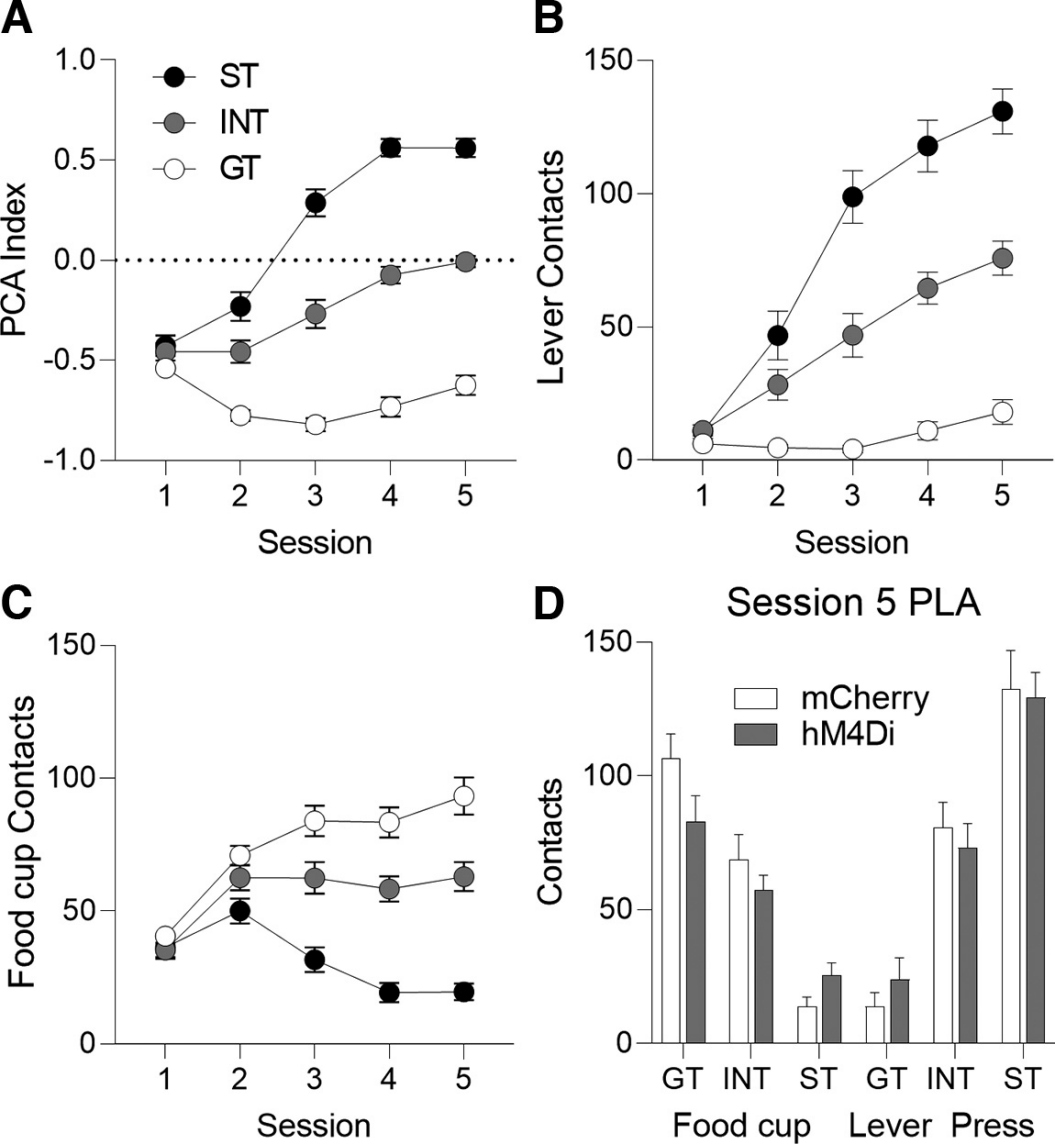
Pavlovian Lever Autoshaping (PLA) data. Pavlovian conditioned approach (PCA) index (***A***), lever contacts (***B***), and food cup contacts (***C***) across 5 d of training. ***D***, Lever and food cup contacts on the fifth day of PLA across tracking groups and between viral conditions. There were no differences between viral conditions within each tracking group (*p*s* *>* *0.05). Data are mean ± SEM. ST = Sign-tracking rats. INT = Intermediate rats. GT = Goal-tracking rats.

Before devaluation testing, we trained rats on five sessions of PLA to determine tracking phenotype by examining lever-directed and food-cup-directed behaviors. Tracking phenotype is determined by a rat’s PCA index (Fig. 1*A*; for calculation, see Materials and Methods) on the last two sessions of PLA before devaluation testing and is based on the difference between the number of lever presses (Fig. 1*B*) and food cup pokes (Fig. 1*C*) as well as the difference score for latency and probability to engage with the lever and food cup. In Table 1, we report the main effects and interactions for the autoshaping data using six separate mixed-design repeated measures ANOVAs. Tracking group (ST, INT, GT) was the between-subjects factor, and session (1–5) was the within-subjects factor. To confirm there were no differences between viral groups (mCherry, hM4Di) within each tracking group (ST, INT, GT) before devaluation testing, we analyzed number of lever and food cup contacts during the 5th session of PLA. We found no main effects of virus nor virus × tracking group interactions (*F*s* *<* *2.89, *p*s* *>* *0.05; Fig. 1*D*).

**Table 1:** Repeated measures ANOVA for PLA across all tracking groups during limited training (sessions 1–5)

Effect	Degrees of freedom	Contact		Latency		Probability	
		*F*	*p*	*F*	*p*	*F*	*p*
Lever presses							
Session	(4,288)	**83.92**	**<0.001**	**144.89**	**<0.001**	**122.18**	**<0.001**
Tracking group	(2,72)	**73.03**	**<0.001**	**84.35**	**<0.001**	**88.50**	**<0.001**
Session × tracking group	(8,288)	**21.36**	**<0.001**	**26.93**	**<0.001**	**19.00**	**<0.001**
Food cup pokes							
Session	(4,288)	**24.14**	**<0.001**	**37.98**	**<0.001**	**25.01**	**<0.001**
Tracking group	(2,72)	**35.26**	**<0.001**	**51.09**	**<0.001**	**39.81**	**<0.001**
Session × tracking group	(8,288)	**19.35**	**<0.001**	**18.34**	**<0.001**	**12.48**	**<0.001**

Bold numbers indicate *p* < 0.05.

### Satiety-induced outcome devaluation after limited training

**Figure 2.**
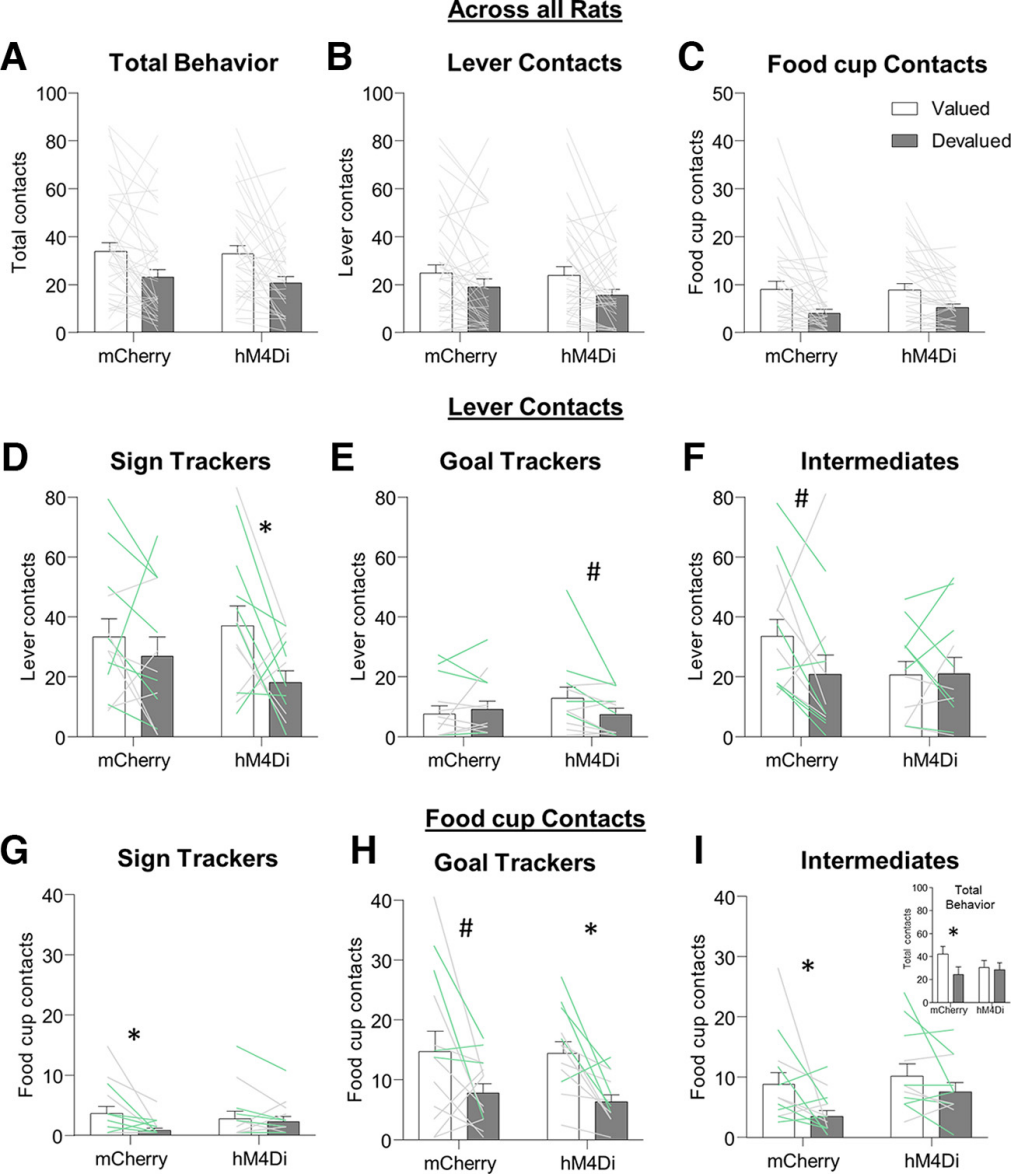
Specific satiety-induced outcome devaluation in Sign-trackers (ST), Goal-trackers (GT), and intermediate (INT) rats. Data are represented as within-subject individual data (lines; green and gray lines represent females and males, respectively) and group averages (bars, mean ± SEM). Overall main effects of outcome value on (***A***) total behavior (lever + food cup contacts) and separately on lever contacts (***B***) and food cup contacts (***C***), with no main effects of virus nor interaction (*p*s* *>* *0.05). ***D–F***, For lever contacts, we observe a virus × tracking × outcome value interaction (see Results), and *post hoc* analysis indicate intact (mCherry) ST and GT show devaluation insensitivity, while intact (mCherry) INT are marginally devaluation sensitive (*t*_(12)_ = 2.07, *p *=* *0.061). While ST and GT rats with BLA-aIC inhibition were devaluation sensitive (ST hM4Di: *t*_(12)_ = 2.63, *p *=* *0.022; GT hM4Di: *t*_(11)_ = 2.12, *p *=* *0.057), INT rats with BLA-aIC inhibition were devaluation insensitive (INT hM4Di: *t*_(10)_ = −0.10, *p *=* *0.926). ***G–I***, For food cup contacts we observe a main effect of outcome value, and our a priori planned comparisons confirm devaluation sensitivity in both GT viral groups (***H***; GT mCherry marginal: *t*_(12)_ = 2.08, *p *=* *0.059; GT hM4Di *t*_(12)_ = 3.94, *p *=* *0.002) and ST and INT mCherry groups (ST: *t*_(12)_ = 2.44, *p *=* *0.031; INT: *t*_(12)_ = 2.38, *p *=* *0.035), but not in the ST and INT hM4Di groups (*p*s* *>* *0.1). ***I***, Inset, Total behavior (sum lever and food cup contacts for intermediate rats). Planned comparisons show intact INT are devaluation sensitive (INT mCherry: *t*_(12)_ = 2.59, *p *=* *0.024) but INT rats with BLA-aIC inhibition were devaluation insensitive (INT hM4Di *t*_(10)_ = 0.359, *p *=* *0.727); *p <* *0.05, #*p *=* *0.06.

We examined whether inactivation of BLA-aIC altered Pavlovian outcome devaluation independent of tracking group (Fig. 2*A*). We first analyzed total behavior (sum of lever and food cup contacts) using the between-subjects factor of virus (mCherry, hM4Di) and within-subjects factor of devaluation (valued, devalued). We observe a main effect of devaluation (*F*_(1,73)_ = 22.67, *p *=* *0.0001) but no other main effects or interactions (*F*s* *<* *0.2, *p*s* *>* *0.5). Next, we repeated the same analysis separately on lever (Fig. 2*B*) and food cup (Fig. 2*C*) behaviors, and found a similar result: main effect of devaluation (lever contacts: *F*_(1,73)_ = 10.34, *p *=* *0.002; food cup contacts: *F*_(1,73)_ = 24.94, *p *<* *0.00001) but no other main effects or interactions (*F*s* *<* *0.6, *p*s* *>* *0.4). Without considering tracking differences in Pavlovian approach, it appears as though BLA-IC pathway inhibition has no effect on responding to cues when outcome value changes.

However, we designed the study to determine whether tracking groups uniquely used the BLA-aIC pathway to drive their differential devaluation sensitivities. Thus, we included tracking phenotype as a between-subjects factor and separately analyzed lever and food cup contacts, the dominant behaviors of sign and goal trackers, respectively. For lever contacts (Fig. 2*D–F*), we observe a virus × tracking group × outcome value interaction (*F*_(2,69)_ = 3.28, *p *=* *0.044) and main effects of tracking group (*F*_(2,69)_ = 12.12, *p* = 0.00003) and outcome value (*F*_(1,69)_ = 10.45, *p *=* *0.002), but no main effect of virus or any other interaction (*F*s* *<* *2.2, *p*s* *>* *0.1). *Post hoc* analyses indicate that intact ST mCherry rats show no difference in lever contacts to valued and devalued conditions (*t*_(12)_ = 1.05, *p *=* *0.316; Fig. 2*D*); however, ST hM4Di expressing rats show greater lever approach to the valued compared with devalued condition (*t*_(12)_ = 2.63, *p *=* *0.022). While GT rats show very low levels of lever approach (i.e., ST behavior), they showed a similar pattern of results with BLA-aIC inactivation (GT mCherry: *t*_(12)_ = −0.75, *p *=* *0.466; GT hM4Di: *t*_(11)_ = 2.12, *p *=* *0.057; Fig. 2*E*). To our surprise, intact INT mCherry rats showed marginally greater lever contacts to valued compared with devalued conditions (*t*_(12)_ = 2.07, *p *=* *0.061; Fig. 2*F*) and pathway inactivation in INT hM4Di rats made lever contacts devaluation insensitive (*t*_(10)_ = −0.10, *p *=* *0.926). These data suggest that for the extreme ends of the tracking continuum (ST and GT rats), disrupting communication between BLA and aIC makes lever approach (i.e., ST behavior) more sensitive to current outcome value. In contrast, rats displaying a mix of lever and food cup approach (INT rats), BLA-aIC inactivation makes lever approach less sensitive to current outcome value. Indeed, an analysis that combines ST and GT lever contact data (i.e., the PCA continuum extremes, STGT group) and compares it to lever data from INT supports these conclusions, with a tracking (STGT, INT) × virus × outcome value interaction (*F*_(1,71)_ = 6.187, *p *=* *0.015). Additionally, an analysis on lever latency data (Table 2) showed consistency with the lever contact data, with a virus × tracking group × outcome value interaction and main effects of tracking group and outcome value, but no main effect of virus or any other interaction (*F*s* *<* *1.6 *p*s* *>* *0.2). In addition, for intermediate lever latency data, we observe a value × virus interaction (*F*_(1,22)_ = 6.006, *p *=* *0.023). *Post hoc*s indicate intact INT rats were slower to respond at the lever when the outcome was devalued (valued: 4.82 ± 1.94; devalued: 6.72 ± 2.09; *p *=* *0.005), and BLA-aIC inactivation in INT rats eliminated this latency difference between devaluation conditions (valued: 5.90 ± 2.59; devalued: 5.69 ± 2.86; *p *>* *0.5).

**Table 2.** Repeated measures ANOVA for latency to lever press or food cup pokes during outcome devaluation across tracking groups and between virus

Effect	Degrees of freedom	Lever presses		Food cup pokes	
		*F*	*p*	*F*	*p*
Virus	(1,69)	0.18	0.670	3.57	0.063
Tracking	(1,69)	**12.29**	**<0.001**	**15.77**	**<0.001**
Outcome value	(1,69)	**14.92**	**<0.001**	**28.17**	**<0.001**
Virus × tacking	(2,69)	0.25	0.782	0.28	0.758
Virus × outcome value	(1,69)	0.078	0.781	0.001	0.977
Tracking × outcome value	(2,69)	1.56	0.217	**3.16**	**0.049**
Virus × tracking × outcome value	(2,69)	**3.38**	**0.040**	1.09	0.343

Bold numbers indicate *p* < 0.05.

For food cup contacts (Fig. 2*G–I*), we observe a main effect of outcome value (*F*_(1,69)_ = 27.03, *p *<* *0.00001) and tracking group (*F*_(2,69)_ = 21.53, *p *<* *0.00001) and outcome value × tracking group interaction (*F*_(2,69)_ = 4.20, *p *= 0.019). While we did not observe a three-way interaction for food cup contacts, our a priori hypothesis was that the preferred response (i.e., food cup contact) in GT rats would become devaluation insensitive with BLA-aIC pathway inhibition. Contrary to our predictions, both GT mCherry and hM4Di rats showed more food cup contacts to valued compared with devalued conditions (main effect of outcome value, *F*_(1,23)_ = 14.16, *p *=* *0.001; Fig. 2*H*). While the remaining food cup approach data should be interpreted with caution because of low levels or responding and/or a lack of three-way interaction, we also observe a main effect of outcome value in both ST (*F*_(1,24)_ = 6.19, *p *=* *0.02; Fig. 2*G*) and INT groups (*F*_(1,22)_ = 7.56, *p *=* *0.012; Fig. 2*I*). Altogether, the data across tracking groups suggests BLA-aIC inhibition does not affect the devaluation sensitivity of food cup approach (Fig. 2*C*). Because INT rats display similar levels of food cup and lever approach, Figure 2*I*, inset, shows total approach (lever + food cup) for INT rats.

**Figure 3.**
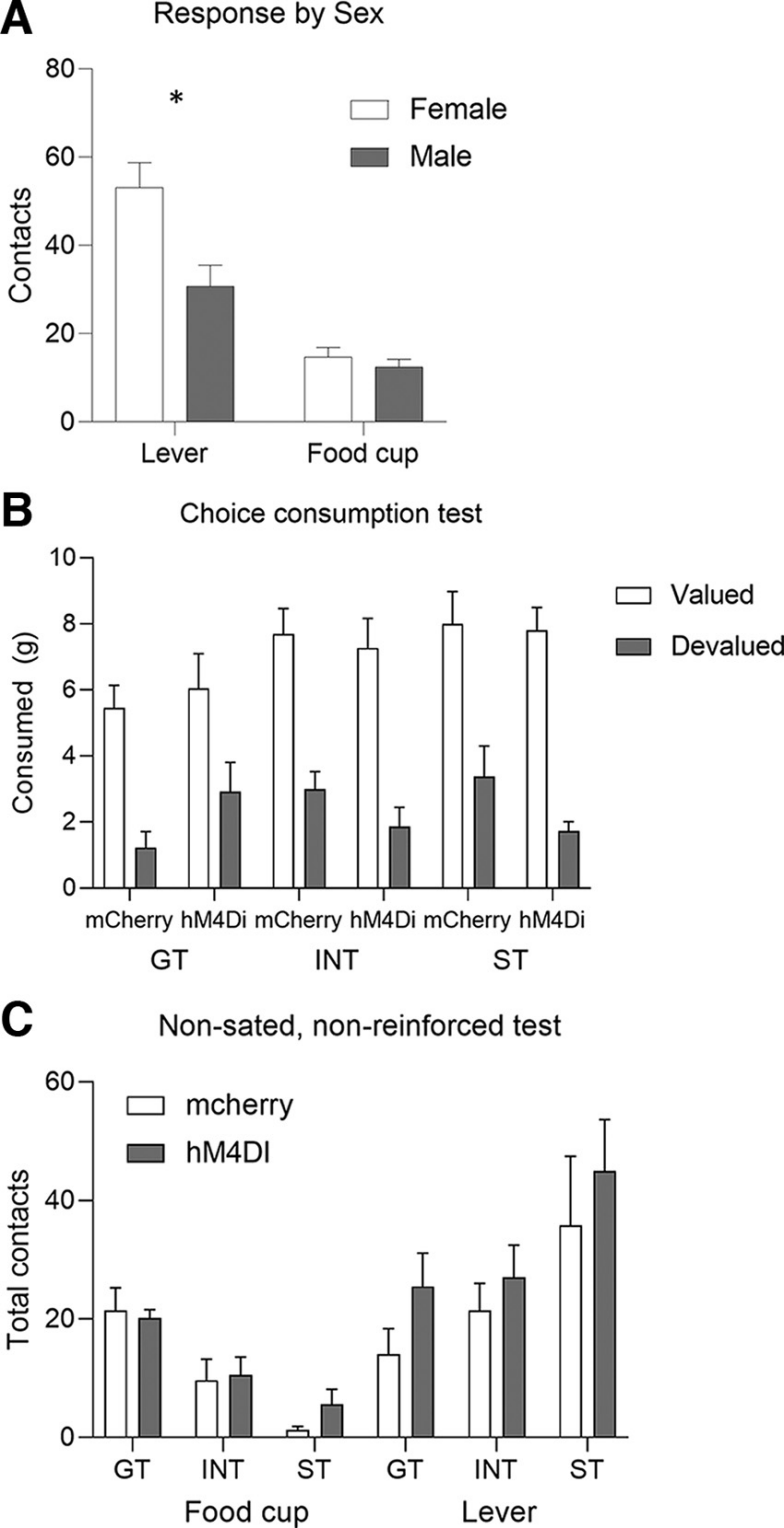
***A***, During outcome devaluation, we observed a response × sex interaction, and *post hoc* analysis show females perform more lever contacts (i.e., ST responses) than males during outcome devaluation (*t*_(73)_ = 3.077, *p *=* *0.003), independent of outcome value or viral condition. There are no differences in food cup contacts. ***B***, We found no differences between tracking or viral groups during the postoutcome devaluation consumption choice test, indicating neither tracking nor BLA-aIC inhibition affected choice to consume the valued over the devalued outcome. ***C***, We found no effects of BLA-aIC inactivation on food cup or lever contacts during a nonsated, nonreinforced test.

Because we used both males and females in this study, we also analyzed the data using Sex instead of Tracking as a factor. The ANOVA including between-subject factors of sex and virus and within-subject factor of response and outcome value yielded main effect of Sex (*F*_(1,71)_ = 11.93, *p *=* *0.0009), and a response × sex interaction (*F*_(1,71)_ = 5.95, *p *=* *0.017), but no other main effects or interactions. Consistent with prior studies, we observe greater levels of lever approach in females compared with males (*t*_(73)_ = 3.077, *p *=* *0.003; Fig. 3*A*).

**Figure 4.**
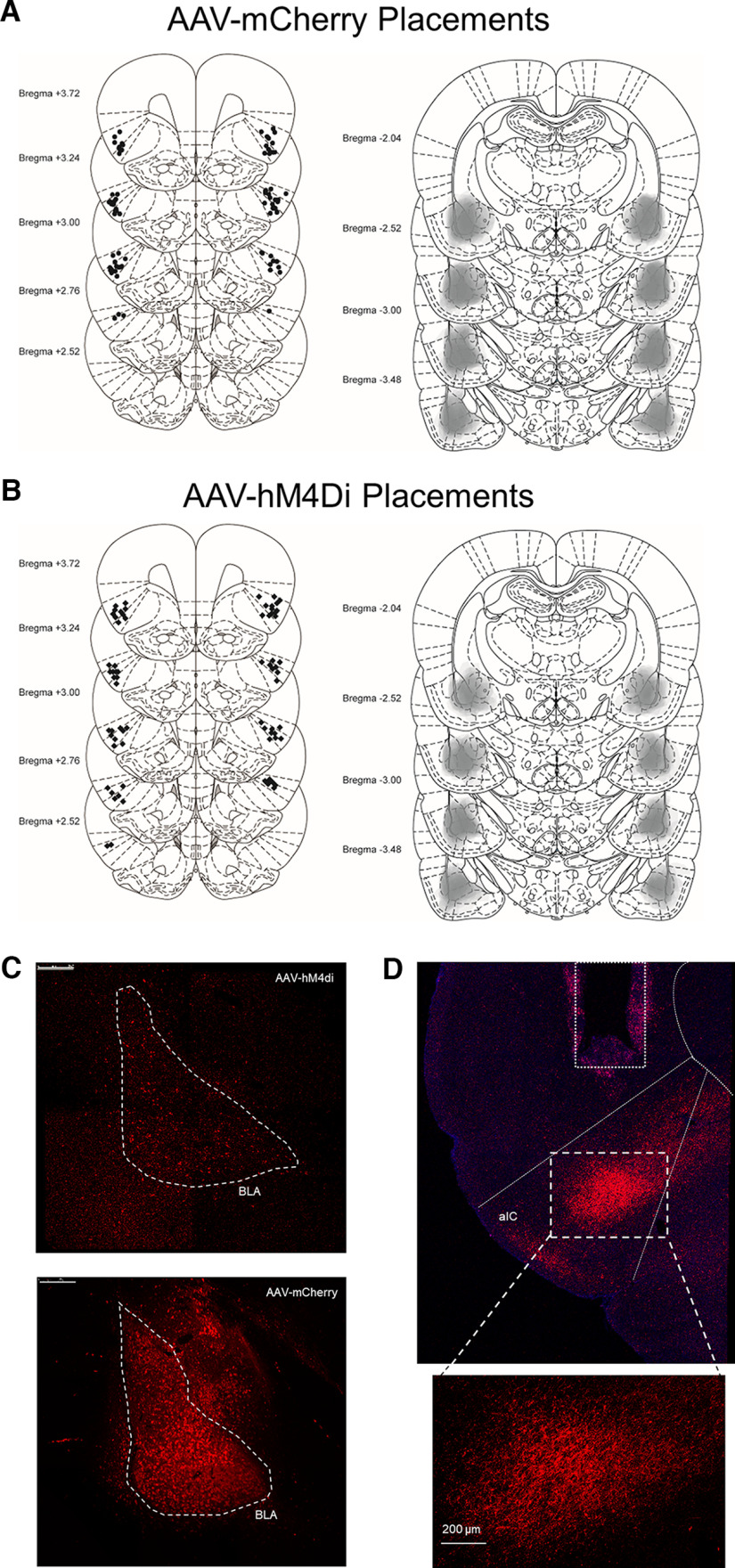
Histologic verification of cannulae in the anterior insular cortex (aIC) and viral expression in the basolateral amygdala (BLA). We implanted bilateral aIC cannulae (***A***, left; ***B***, left) and infused viral constructs into the BLA (***A***, right; ***B***, right; Paxinos and Watson, 2007). Representative images of hM4Di (***C***, top), and mCherry (***C***, bottom). BLA outlined in white dashed lines. Scale bar: 250 μm. ***D***, Representative image of mCherry terminals within the aIC, outlined, and magnification of the terminals (***D***, bottom). Scale bar: 200 μm. Rectangle above aIC indicates cannula placement.

### Consumption, choice test, and nonsated probe test

We sated rats on either chow (valued) or pellets (devalued) before devaluation probe test. We found no differences in the amount of food consumed between tracking groups or virus conditions (*F*s* *<* *2.9, *p*s* *>* *0.09). To confirm devaluation of the sated food, we gave rats a choice test between the food they were sated on and the other food. Rats consumed less of the food they were sated on and more of the alternative food (Fig. 3*B*), verified by a main effect of choice (*F*_(1,69)_ = 169.38, *p *<* *0.00001), with no main effects of tracking or virus (*F*s* *<* *2.6, *p*s* *>* *0.07) and no interactions (*F*s* *<* *2.14, *p*s* *>* *0.1).

To examine whether inactivation of BLA-aIC altered lever or food cup approach independent of specific satiety, we conducted a nonsated, nonreinforced test. In a mixed ANOVA with between-subject factors of virus and tracking and within subjects factor of response, there was no main effect of virus nor interactions with virus (*F*s* *<* *2.5, *p*s* *>* *0.1; Fig. 3*C*), suggesting that BLA-aIC inactivation did not affect lever or food cup behaviors when rats were not sated.

## Discussion

In the current study, we examined whether communication from the BLA to the aIC is necessary for Pavlovian specific satiety-induced outcome devaluation. While we did not observe an overall effect of the manipulation on outcome devaluation, when we include tracking phenotype in the analysis, we observe tracking-specific effects of BLA-aIC pathway inhibition on devaluation sensitivity. Consistent with previous findings, we find that food cup behavior of intact GT rats is devaluation sensitive while lever-directed behavior of intact ST rats is devaluation insensitive (Morrison et al., 2015; Nasser et al., 2015; Patitucci et al., 2016; Smedley and Smith, 2018; Keefer et al., 2020; Kochli et al., 2020). For the ST response (i.e., lever-directed behaviors), BLA-aIC inhibition promoted devaluation sensitivity in ST (and to small extent in GT) rats, but devaluation insensitivity in INT rats. BLA-aIC inhibition had minimal effect on devaluation sensitivity of the GT response (i.e., food-cup-directed behaviors). Yet, the qualitatively consistent effects of BLA-aIC inhibition on both lever and food cup behavior of INT rats suggest the BLA-aIC pathway may be promoting behavioral flexibility in these rats, while the same pathway supports rigid ST behaviors for rats on extreme ends of the tracking continuum.

Importantly, BLA-aIC communication is necessary for full expression of ST and GT behaviors (Nasser et al., 2018). Contralateral disconnection of the BLA and aIC with baclofen/muscimol decreased food cup approach (in GT rats) and increased the latency to contact both the food cup (in GT rats) and lever (in ST rats), seemingly disrupting both GT and ST behaviors. Based on findings that GT rats are devaluation sensitive but ST rats are not, we hypothesized that the content of the associative information encoded in BLA-aIC pathway may differ between ST and GT rats. We predicted that GT rats use BLA-aIC to encode flexible stimulus-outcome (S-O) associations that are necessary for outcome devaluation sensitivity (Holland, 1998). Because ST rats rely on communication between BLA and aIC to promote vigor of the ST response that is insensitive to devaluation, we predicted that ST rats use BLA-aIC to encode rigid stimulus-response (S-R) associations that are insensitive to outcome devaluation (Holland and Rescorla, 1975; Nasser et al., 2018). We did not observe the hypothesized effect of BLA-aIC inactivation on Pavlovian S-O associations in GT rats, but we did observe disruption of S-O-dependent devaluation sensitivity in INT rats with BLA-aIC pathway inactivation. Notably, both lever-directed and food-cup-directed behaviors were devaluation sensitive in intact INT rats. BLA-aIC inactivation made INT rats devaluation insensitive, yet unmasked devaluation sensitivity of lever approach in both GT and ST rats. While there is increasing evidence that distinct neural circuits support ST and GT (Flagel et al., 2011; Nasser et al., 2018; Haight et al., 2020; Pribut et al., 2022), the present findings suggest the content of the associative information encoded within the same neural pathway can vary depending on individual phenotype. Prior studies support the later explanation, with BLA to nucleus accumbens and prelimbic prefrontal cortex to paraventricular thalamus inactivation showing opposite behavioral effects in sign and goal trackers (Campus et al., 2019; Kochli et al., 2020).

Consistent with our predictions for ST rats, we do find evidence for rigid encoding of S-R associations in the BLA-aIC pathway, as inhibiting this pathway makes ST rats sensitive to outcome devaluation. Both the BLA and aIC are heavily implicated in appetitive motivational processes (see Izquierdo, 2017; Parkes et al., 2018; Centanni et al., 2021). The BLA is necessary for incentive processes, particularly for the expression of ST (Chang et al., 2012), and other incentive learning processes, such as second-order conditioning (Hatfield et al., 1996; Setlow et al., 2002; Holland, 2016) and conditioned reinforcement (Parkinson et al., 2001; Burke et al., 2007; for review, see Wassum and Izquierdo, 2015; Keefer et al., 2021). This finding is consistent with a prior study reporting that inhibition of BLA-nucleus accumbens core communication also makes ST rats sensitive to outcome devaluation (Kochli et al., 2020). Together studies suggest an overabundance of rigid appetitive encoding in BLA projections makes ST rats insensitive to outcome devaluation (Nasser et al., 2015; Kochli et al., 2020).

Perhaps most surprising is our failure to observe effects of BLA-aIC pathway inactivation in GT rats; if anything, GT rats expressing the inhibitory DREADD construct showed qualitatively stronger devaluation than intact rats, suggesting that encoding in BLA-aIC may also support rigid S-R associations in GT rats, similar to what we observe in ST rats.

We consider these findings in the context of a previous study that showed temporally specific engagement of the BLA and IC during specific satiety outcome devaluation. In a series of experiments, Parkes and Balleine (2013) demonstrated the BLA is necessary for updating outcome value during satiation, but not necessary for the retrieval of this new value during test, findings consistent with BLA’s role in Pavlovian devaluation (Hatfield et al., 1996; Setlow et al., 2002; Johnson et al., 2009; West et al., 2012). Then, they demonstrated the IC is necessary for the retrieval of the new outcome value at test, but not for the initial encoding of the outcome value during satiety (Parkes and Balleine, 2013; Parkes et al., 2018). Since we sated rats before BLA-aIC inhibition, one possible explanation for lack of effects in GT rats is that the BLA may have already updated aIC on the new outcome value so that it was successfully retrieved at test to support devaluation sensitivity (see Piette et al., 2012). Inhibition of the BLA-aIC pathway before satiation may result in outcome devaluation insensitivity in otherwise devaluation sensitive behaviors, an avenue for future research. Interpreting the improvement in flexibility in ST rats with BLA-aIC inactivation within this framework suggests the rigid S-R association persist beyond the encoding stage (satiety) and is dominant at time of retrieval. The opposite appears to be the case in INT rats, in which BLA-aIC communication of updated outcome value (i.e., flexible S-O association) is necessary for devaluation sensitivity at time of memory retrieval.

Unlike a prior study, disrupting communication between BLA and aIC did not disrupt ST and GT. BLA-aIC inactivation disrupted bidirectional communication between the BLA and aIC during a reinforced lever autoshaping test (Nasser et al., 2018). In contrast, the current test was under extinction conditions and inhibited direct communication from BLA to aIC, while leaving communication from the aIC to BLA intact. The reinforced versus extinction conditions may account for the difference. However, it may be the case that prior effects of contralateral inactivation on GT could be because of aIC to BLA communication. In support of this hypothesis, several studies have probed the necessity of the neighboring OFC. These prior OFC lesion studies also lesioned the aIC, leading us to predict that some behaviors attributed to the OFC may in part be because of aIC damage. These studies concluded the OFC/aIC is necessary to retrieve and express the value of the outcome and associated cues during periods of behavioral flexibility (Gallagher et al., 1999; Pickens et al., 2003, 2005; Ostlund and Balleine, 2007). Additional studies indicate that communication between the BLA and OFC are necessary for outcome devaluation (Baxter et al., 2000; Fiuzat et al., 2017), and direct projections from the BLA to OFC and from the OFC to BLA are critical for Pavlovian, but not instrumental, outcome devaluation (Lichtenberg et al., 2017; Malvaez et al., 2019). Similarly, communication between the BLA and OFC is necessary for other behavioral flexibility paradigms, such as over-expectation (Lucantonio et al., 2015), outcome-specific Pavlovian-to-instrumental transfer (Lichtenberg et al., 2017; Sias et al., 2021), reversal learning (Groman et al., 2019), and risky decision-making (Zeeb and Winstanley, 2013; for review, see Keefer et al., 2021).

Altogether, we conclude by suggesting the utility of the PLA procedure for identifying individual differences that elucidate unique pathway contributions to behavioral flexibility. In the present study, ST rats show less rigid approach strategies when BLA-aIC pathway activity is decreased, indicating that rigid reward seeking is supported by BLA-aIC communication. This is of particular relevance to clinical work indicating enhanced BLA-aIC functional connectivity in acutely abstinent smokers and increased cue reactivity in the BLA-aIC network in individuals most vulnerable to nicotine relapse (Janes et al., 2010; Sutherland et al., 2013). Enhanced resting state functional connectivity between BLA and aIC is also associated with greater state and trait anxiety as well as with PTSD (Baur et al., 2013; Centanni et al., 2021; Fonzo et al., 2021). Thus, increased amygdalar-insular connectivity and cue reactivity are commonly implicated in mental health disorders with high rates of comorbidity (Back and Brady, 2008; McCauley et al., 2012; María-Ríos and Morrow, 2020). Consistent with the clinical work, the preclinical data indicate ST rats that engage BLA-aIC to drive rigid, cue-triggered reward seeking also show greater cue-triggered cocaine relapse and enhanced vulnerability in a model of PTSD (Saunders and Robinson, 2010; Morrow et al., 2015). While future work is needed to determine the contribution of BLA-aIC communication to relapse and PTSD vulnerability in rodent models, the ST model has the potential to span the translational gap between rodents and humans to better understand the behavioral and brain circuit contributions to rigid, cue-reactive reward seeking.

## Synthesis

Reviewing Editor: Mark Laubach, American University Decisions are customarily a result of the Reviewing Editor and the peer reviewers coming together and discussing their recommendations until a consensus is reached. When revisions are invited, a fact-based synthesis statement explaining their decision and outlining what is needed to prepare a revision will be listed below. The following reviewer(s) agreed to reveal their identity: Kurt Fraser, Stephanie Groman.

Your manuscript was reviewed by two experts and their comments are below. Please revise the manuscript to address all points that were raised. Thank you for sending your research to eNeuro!

Reviewer #1

In this report the authors assess the contribution of a pathway from the basolateral amygdala to the anterior insula in promoting goal-directed Pavlovian responding. The authors do so by making use of the ability to identify rats as sign- or goal-trackers following Pavlovian conditioned approach training where a lever predicts delivery of a food pellet. Following training, rats with either mCherry or the inhibitory DREADD hM4Di were infused with the DREADD ligand CNO in the anterior insula immediately following satiation with either the reward earned during the task or the chow they normally consume (the rats were lightly-food restricted and as a result would eat in this window). The authors find that inactivation of this pathway in sign-tracking rats reduced responding in a non-reinforced test session selectively when sated on the reward normally earned. There were no prominent effects on goal-tracking (the rats maintained devaluation-sensitive responding). Interestingly, in intermediate rats this manipulation appears to have made their responding insensitive, the opposite of the effect observed in sign-trackers. I found the results intriguing and adding to a growing literature attempting to understand the neural circuits important for Pavlovian responding in cases where the content of learning transforms the conditioned response. I found the experiments straightforwardly presented but have some comments regarding the discussion and presentation of the findings and felt that the authors could dive deeper into the data to clarify some potential interesting findings.

1. The title implies that the inverse experiment was performed in sign-tracking rats, e.g. stimulating the pathway makes them insensitive. I would suggest the re-framing of the paper as inactivation of this pathway restores or makes sign-tracking sensitive to outcome devaluation.

2. In older literature the anterior insula was frequently labeled as the orbitofrontal cortex and the authors here reference literature from some of these studies as evidence to support a specific role for the anterior insula, yet still refer to these as the orbitofrontal cortex which led to some confusion on my first and second readings, e.g. why would orbitofrontal findings be relevant for thinking about the insula more than prelimbic or anterior cingulate findings. Some further explanation of why a need to focus on this projection with regard to these discrepancies is needed. The paper concludes by summarizing findings on amygdala and orbitofrontal cortex interactions, for example, which seem tertiary to the primary results here.

3. I don’t quite understand the argument presented in the discussion (lines 326-332) that this exact same neural pathway encodes outcome-specific motivational information in goal-trackers and outcome-insensitive motivational information in sign-trackers. How could activity in this exact same projection encode and promote these two totally distinct motivational processes? Isn’t it perhaps equally or more likely that goal-tracking relies on distinct circuits from those in sign-trackers? I felt that this argument could be further expanded on - particularly if the authors are making the argument that the same circuits are engaged in both sign- and goal-tracking yet the actual content within each of these circuits is different. There is increasing evidence in other reports that there are distinct “sign-tracking” circuits and “goal-tracking” circuits so the authors could more clearly frame their argument in this regard.

4. Is the magnitude of the devaluation effect related to the individual PCA index score or responding on the final session before devaluation?

5. In a previous paper disconnection of this pathway had multifacted effects on behavior beyond the number of contacts at the lever or food cup. Do the authors have any findings with respect to the latency or probability of responding? This would help clarify the results in intermediate rats as being potentially from indecisiveness on trial-to-trial which response to make and drive lower responses overall compared to their mCherry controls.

6. Why does behavior more generally appear to be increased lever contacts for all groups in the non-sated, non-reinforced test? Goal-trackers and intermediates seem to even be making more lever contacts than food cup entries.

7. Did devaluation and inactivation reduce responding for sign-tracking rats relative to their mCherry controls or is it solely a within-subjects effect? I could not find evidence for such a contrast being performed.

8. A trial by trial analysis would be helpful to understand and parse whether the effects of inactivation are accelerating extinction throughout the non-reinforced session or if these effects are present on the first trial (a purer assessment of the incorporation of updated outcome value in Pavlovian motivation).

9. Representative histology for the cannula tracts in the anterior insula with mCherry-tagged axon terminals would be helpful.

10. Were misses systematic in anyway that would lead to targeting of the lateral orbitofrontal cortex?

11. Was Sex included as a fourth factor in the model or only separately from response strategy? It would be informative to explore ways to incorporate this into the statistical approach and also to indicate on the individual traces which animal is male or female with different colors or styles.

Reviewer #2

The paper titled “Basolateral amygdala to insular cortex activity makes sign tracking behavior insensitive to outcome value” describes a study examining the role of the BLA aIC pathway in outcome devaluation of sign tracking (ST)/goal tracking (GT). The authors used DREADDs to selectively express the inhibitory DREADD in amygdala neurons and then infused CNO into the aIC at time of test to inhibit amygdala input into the aIC. Following the viral and cannulae placement surgeries, a pavlovian lever autoshaping task was used to characterize rats as having a sign tracker, goal tracker or intermediate phenotype. The authors then conducted an outcome devaluation test (outcome specific satiety) following CNO administration. The authors hypothesized that inactivating the BLA aIC pathway would make GT rats insensitive to outcome devaluation. ST rats, however, are insensitive to outcome devaluation at baseline so the authors hypothesized that inhibition of BLA aIC would generally reduce behavior. The authors found results that were almost completely opposite to what they hypothesized. BLA aIC inhibition made ST rats more sensitive to devaluation but did not alter behavior of GT rats in the devaluation test. The paper is very well written - rationale for experiment is well described, the methods and materials were easy to follow, and the analyses were very clear - and the results are interesting. My only comments to the authors are with respect to their interpretation of the data.

First, I’m not sure I understand the argument that the BLA aIC circuit in the INT rats controls behavioral flexibility, but “rigid sign-tracking behaviors” in the ST and GT rats. Inhibition of the BLA aIC circuit did not alter the behaviors of GT rats (based on the statistics reported in the paper) so it seems to me that GT behaviors are most likely mediated by a different circuit (e.g., aIC BLA) that was not investigated. Perhaps the authors can clarify their argument.

Second, the authors caution the reader about interpreting low levels of responding (Pg 11) but then use this data to interpret their effects (lever contacts in GT rats and food cup contacts in ST). I’m not opposed to this, but I think the authors should note in the discussion that some of their interpretations are based on responses going from 4 to 2 (Fig 2H).

Third, I was hoping there would be more of a discussion about how the behavioral impact of circuit-specific manipulations varied across ST, GT, and INT rats. The abstract suggests that this is the major finding of the paper, but it is glossed over in the discussion. I agree with the authors that this a major finding and would like see more about the clinical relevance of this result.

Minor comments:

Figure 2 - I understand why the authors moved GTs to the first position on the third row, but it was a bit confusing. I recommend swapping G and H.
